# Supplementary material for: Microbial co-occurrences on catheters from long-term catheterized patients
Source: Nat Commun. 2024 Jan 2;15:61. doi: 10.1038/s41467-023-44095-0 (PMC10762172; doi:10.1038/s41467-023-44095-0)
Supplement: Supplementary file 1 — Supplementary Information [file 41467_2023_44095_MOESM1_ESM.pdf]

Supplementary Information

Microbial co-occurrences on catheters from long-term catheterized patients

Taylor M. Nye[1†], Zongsen Zou[1†], Chloe L.P. Obernuefemann[1], Jerome S. Pinkner[1], Erin Lowry[1], Kent Kleinschmidt[1], Karla Bergeron[2], Aleksandra Klim[2,] Karen W. Dodson[1], Ana Flores-Mireles[3], Jennifer N Walker[4], Daniel Garrett Wong[2], Alana Desai[2], Michael G. Caparon[1*], and Scott J. Hultgren[1*]

[†]These authors contributed equally.
*Address correspondence to Scott J. Hultgren, hultgren@wustl.edu and Michael G. Caparon, caparon@wustl.edu

[1] Department of Molecular Microbiology and Center for Women's Infectious Disease Research, Washington University School of Medicine, Saint Louis, MO 63110-1093, USA.[2]Department of Surgery, Division of Urologic Surgery, Washington University School of Medicine, Saint Louis, MO 63110, USA [3] Department of Biological Sciences, University of Notre Dame, Notre Dame, IN 46556, USA.[4]Department of Microbiology and Molecular Genetics, McGovern Medical School, The University of Texas Health Science Center, Houston, TX 77030, USA.

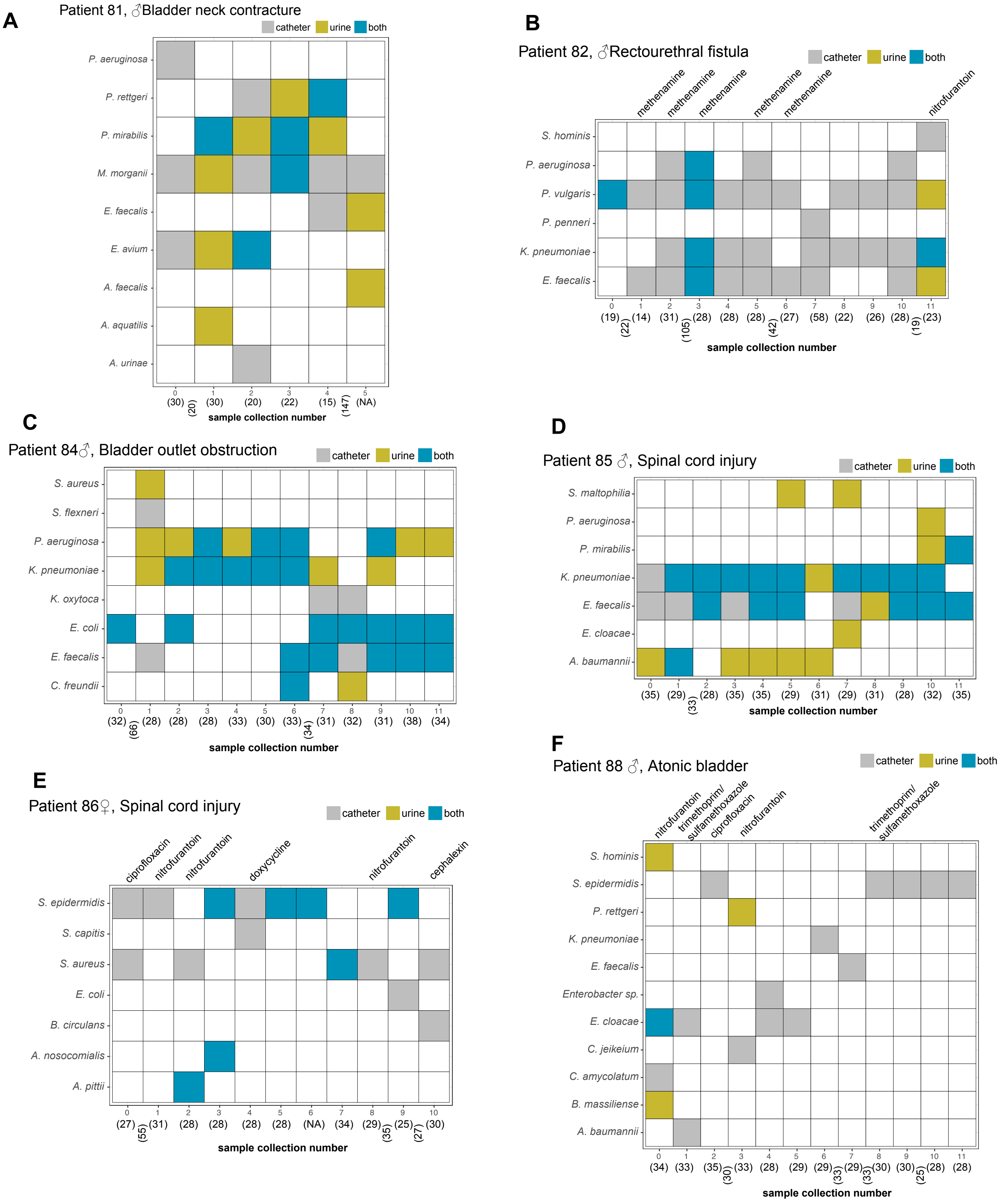

A
Patient 81, ♂Bladder neck contracture
catheter urine both
P. aeruginosa
P. rettgeri
P. mirabilis
M. morganii
E. faecalis
E. avium
A. faecalis
A. aquatilis
A. urinae
0 1 2 3 4 5
(30) (20) (30) (20) (22) (15) (147) (NA)
sample collection number
B
Patient 82, ♂Rectourethral fistula
catheter urine both
methenamine
methenamine
methenamine
methenamine
methenamine
nitrofurantoin
S. hominis
P. aeruginosa
P. vulgaris
P. penneri
K. pneumoniae
E. faecalis
0 1 2 3 4 5 6 7 8 9 10 11
(19) (22) (14) (31) (105) (28) (28) (28) (42) (27) (58) (22) (26) (28) (19) (23)
sample collection number
C
Patient 84♂, Bladder outlet obstruction
catheter urine both
S. aureus
S. flexneri
P. aeruginosa
K. pneumoniae
K. oxytoca
E. coli
E. faecalis
C. freundii
0 1 2 3 4 5 6 7 8 9 10 11
(32) (66) (28) (28) (28) (33) (30) (33) (34) (31) (32) (31) (38) (34)
sample collection number
D
Patient 85 ♂, Spinal cord injury
catheter urine both
S. maltophilia
P. aeruginosa
P. mirabilis
K. pneumoniae
E. faecalis
E. cloacae
A. baumannii
0 1 2 3 4 5 6 7 8 9 10 11
(35) (29) (33) (28) (35) (35) (29) (31) (29) (31) (28) (32) (35)
sample collection number
E
Patient 86♀, Spinal cord injury
catheter urine both
ciprofloxacin
nitrofurantoin
nitrofurantoin
doxycycline
nitrofurantoin
cephalexin
S. epidermidis
S. capitis
S. aureus
E. coli
B. circulans
A. nosocomialis
A. pittii
0 1 2 3 4 5 6 7 8 9 10
(27) (55) (31) (28) (28) (28) (28) (NA) (34) (29) (35) (25) (27) (30)
sample collection number
F
Patient 88 ♂, Atonic bladder
catheter urine both
nitrofurantoin
trimethoprim/ sulfamethoxazole
ciprofloxacin
nitrofurantoin
trimethoprim/ sulfamethoxazole
S. hominis
S. epidermidis
P. rettgeri
K. pneumoniae
E. faecalis
Enterobacter sp.
E. cloacae
C. jeikeium
C. amycolatum
B. massiliense
A. baumannii
0 1 2 3 4 5 6 7 8 9 10 11
(34) (33) (35) (30) (33) (28) (29) (29) (33) (29) (33) (30) (30) (25) (28) (28)
sample collection number

Supplementary Figure 1. Species identification, catheter dwell times, and antibiotics prescribed to long-term catheterized patients. Sample collection information for Patients 81, 82, 84, 85, 86, and 88 (**A-F).** Collection period is indicated on the x-axis with the duration of the period in parenthesis (days). The duration (days) of missed collection periods due to hospitalization or other causes are listed in vertical parenthesis between collection periods. Species detected within the patient are indicated on the y-axis, with gray denoting the species isolated from the catheter, yellow from the urine, and blue from the catheter and the urine at the given collection point. The antibiotics prescribed over the collection period are indicated at the top of each column. Patients with greater than one collection with at least two different species identified are included.

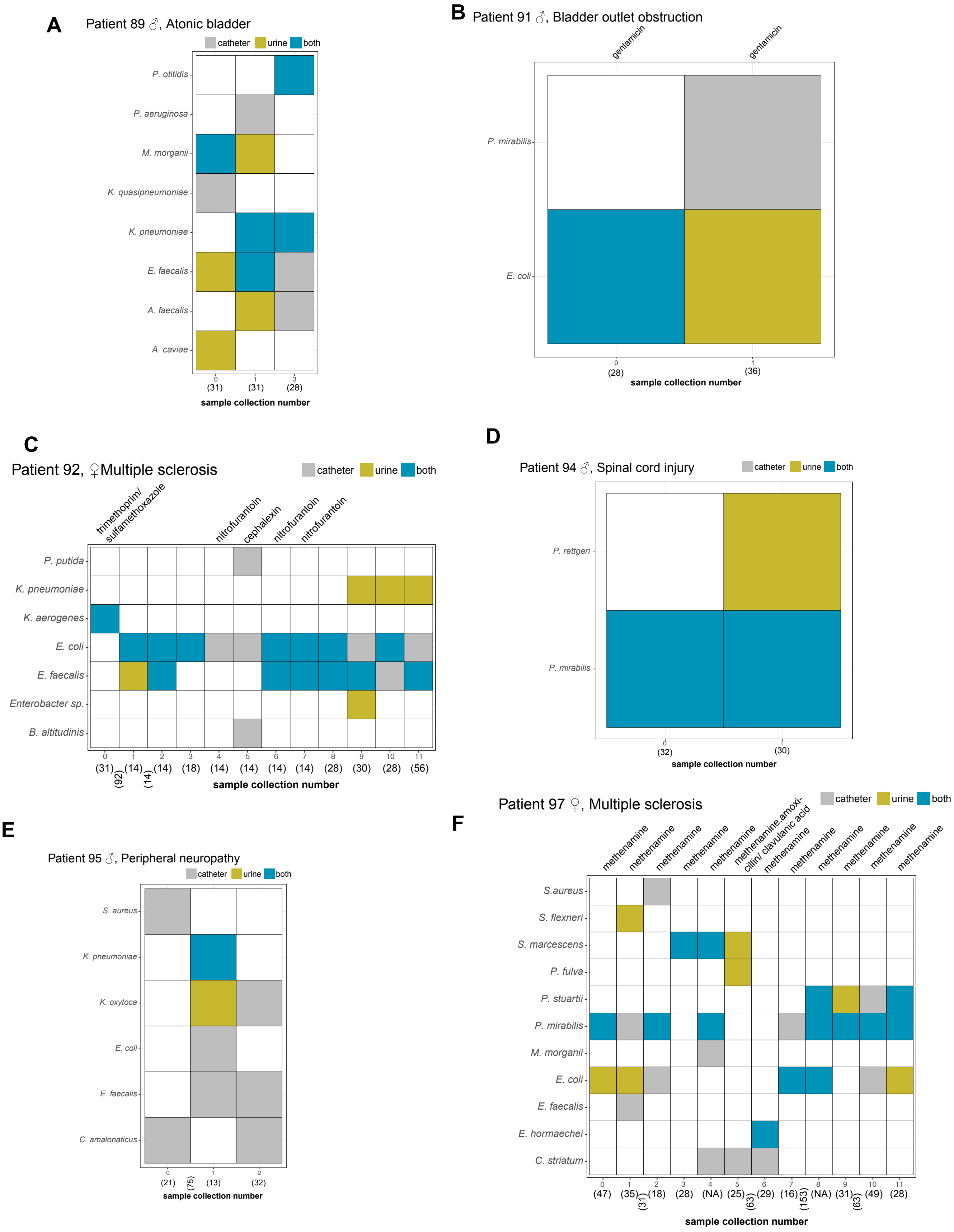
A
Patient 89 ♂, Atonic bladder
catheter
urine
both
P. otitidis
P. aeruginosa
M. morganii
K. quasipneumoniae
K. pneumoniae
E. faecalis
A. faecalis
A. caviae
0
(31)
1
(31)
3
(28)
sample collection number
B
Patient 91 ♂, Bladder outlet obstruction
gentamicin
gentamicin
P. mirabilis
E. coli
0
(28)
1
(36)
sample collection number
C
Patient 92, ♀Multiple sclerosis
catheter
urine
both
trimethoprim/ sulfamethoxazole
nitrofurantoin
cephalexin
nitrofurantoin
nitrofurantoin
P. putida
K. pneumoniae
K. aerogenes
E. coli
E. faecalis
Enterobacter sp.
B. altitudinis
0 1 2 3 4 5 6 7 8 9 10 11
(31) (14) (14) (18) (14) (14) (14) (14) (28) (30) (28) (56)
(92)
(14)
sample collection number
D
Patient 94 ♂, Spinal cord injury
catheter
urine
both
P. rettgeri
P. mirabilis
0
(32)
1
(30)
sample collection number
E
Patient 95 ♂, Peripheral neuropathy
catheter
urine
both
S. aureus
K. pneumoniae
K. oxytoca
E. coli
E. faecalis
C. amalonaticus
0
(21)
(75)
1
(13)
2
(32)
sample collection number
F
Patient 97 ♀, Multiple sclerosis
catheter
urine
both
methenamine
methenamine
methenamine
methenamine
methenamine
methenamine,amoxicillin/ clavulanic acid
methenamine
methenamine
methenamine
methenamine
methenamine
methenamine
S.aureus
S. flexneri
S. marcescens
P. fulva
P. stuartii
P. mirabilis
M. morganii
E. coli
E. faecalis
E. hormaechei
C. striatum
0 1 2 3 4 5 6 7 8 9 10 11
(47) (35) (18) (28) (NA) (25) (29) (16) (NA) (31) (49) (28)
(31)
(63)
(153)
(63)
sample collection number

Supplementary Figure 2. Species identification, catheter dwell times, and antibiotics prescribed to long-term catheterized patients. Sample collection information for Patients 89, 91, 92, 94, 95, and 97 (**A-F).** Collection period is indicated on the x-axis with the duration of the period in parenthesis (days). The duration (days) of missed collection periods due to hospitalization or other causes are listed in vertical parenthesis between collection periods. Species detected within the patient are indicated on the y-axis, with gray denoting the species isolated from the catheter, yellow from the urine, and blue from the catheter and the urine at the given collection point. The antibiotics prescribed over the collection period are indicated at the top of each column. Patients with greater than one collection with at least two different species identified are included.

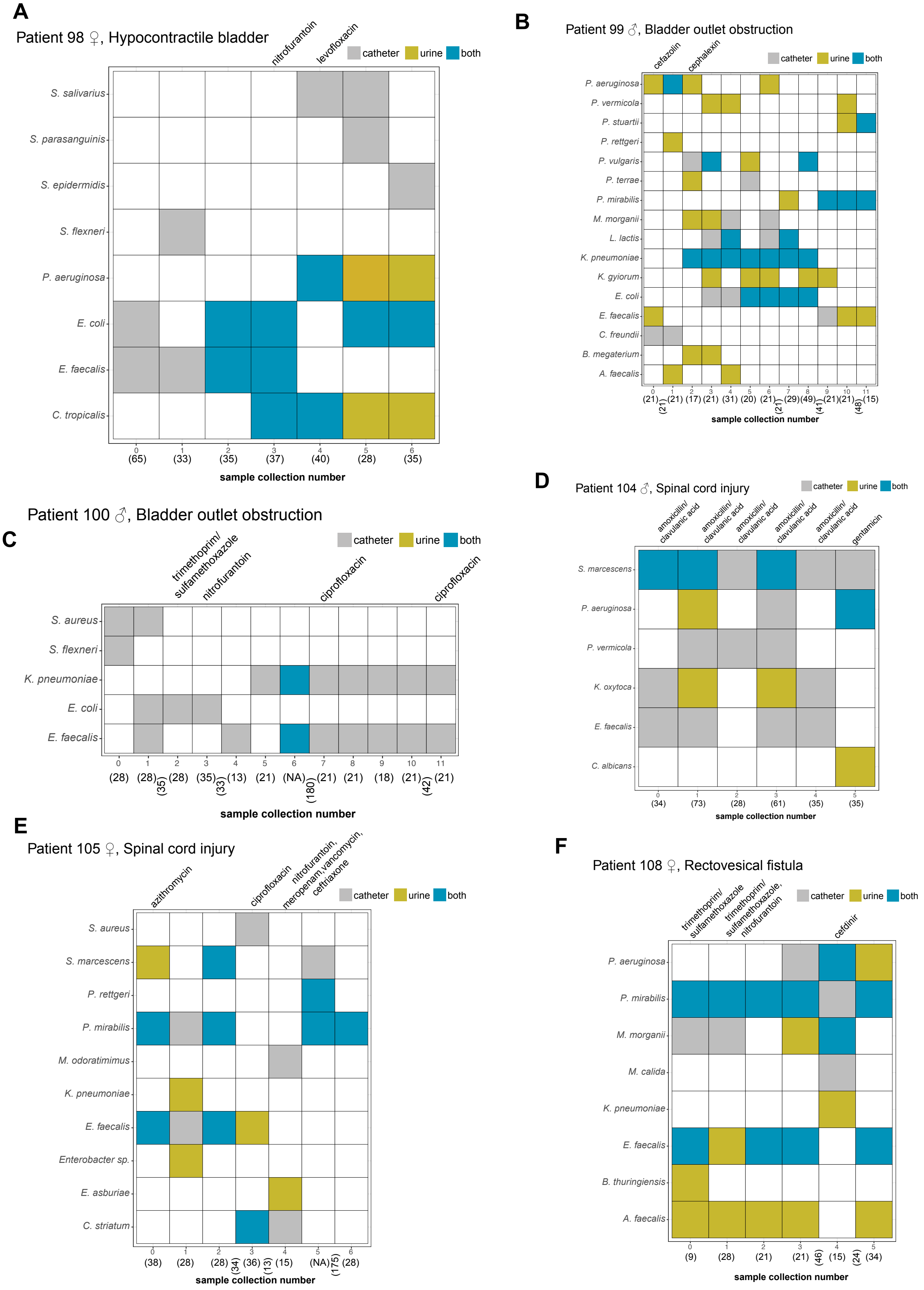
A
Patient 98 ♀, Hypocontractile bladder
nitrofurantoin
levofloxacin
catheter
urine
both
S. salivarius
S. parasanguinis
S. epidermidis
S. flexneri
P. aeruginosa
E. coli
E. faecalis
C. tropicalis
0 1 2 3 4 5 6
(65) (33) (35) (37) (40) (28) (35)
sample collection number
B
Patient 99 ♂, Bladder outlet obstruction
cefazolin
cephalexin
catheter
urine
both
P. aeruginosa
P. vermicola
P. stuartii
P. rettgeri
P. vulgaris
P. terrae
P. mirabilis
M. morganii
L. lactis
K. pneumoniae
K. gyiorum
E. coli
E. faecalis
C. freundii
B. megaterium
A. faecalis
0 1 2 3 4 5 6 7 8 9 10 11
(21) (21) (21) (17) (21) (31) (20) (21) (21) (29) (49) (41) (21) (21) (48) (15)
sample collection number
C
Patient 100 ♂, Bladder outlet obstruction
trimethoprim/
sulfamethoxazole
nitrofurantoin
ciprofloxacin
ciprofloxacin
catheter
urine
both
S. aureus
S. flexneri
K. pneumoniae
E. coli
E. faecalis
0 1 2 3 4 5 6 7 8 9 10 11
(28) (28) (35) (28) (35) (33) (13) (21) (NA) (180) (21) (21) (18) (21) (42) (21)
sample collection number
D
Patient 104 ♂, Spinal cord injury
catheter
urine
both
amoxicillin/
clavulanic acid
amoxicillin/
clavulanic acid
amoxicillin/
clavulanic acid
amoxicillin/
clavulanic acid
amoxicillin/
clavulanic acid
gentamicin
S. marcescens
P. aeruginosa
P. vermicola
K. oxytoca
E. faecalis
C. albicans
0 1 2 3 4 5
(34) (73) (28) (61) (35) (35)
sample collection number
E
Patient 105 ♀, Spinal cord injury
azithromycin
ciprofloxacin
nitrofurantoin,
meropenam,vancomycin,
ceftriaxone
catheter
urine
both
S. aureus
S. marcescens
P. rettgeri
P. mirabilis
M. odoratimimus
K. pneumoniae
E. faecalis
Enterobacter sp.
E. asburiae
C. striatum
0 1 2 3 4 5 6
(38) (28) (28) (34) (36) (13) (15) (NA) (175) (28)
sample collection number
F
Patient 108 ♀, Rectovesical fistula
trimethoprim/
sulfamethoxazole
trimethoprim/
sulfamethoxazole,
nitrofurantoin
cefdinir
catheter
urine
both
P. aeruginosa
P. mirabilis
M. morganii
M. calida
K. pneumoniae
E. faecalis
B. thuringiensis
A. faecalis
0 1 2 3 4 5
(9) (28) (21) (21) (46) (15) (24) (34)
sample collection number

Supplementary Figure 3. Species identification, catheter dwell times, and antibiotics prescribed to long-term catheterized patients. Sample collection information for Patients 98, 99, 100, 104, 105, and 108 (**A-F).** Collection period is indicated on the x-axis with the duration of the period in parenthesis (days). The duration (days) of missed collection periods due to hospitalization or other causes are listed in vertical parenthesis between collection periods. Species detected within the patient are indicated on the y-axis, with gray denoting the species isolated from the catheter, yellow from the urine, and blue from the catheter and the urine at the given collection point. The antibiotics prescribed over the collection period are indicated at the top of each column. Patients with greater than one collection with at least two different species identified are included.

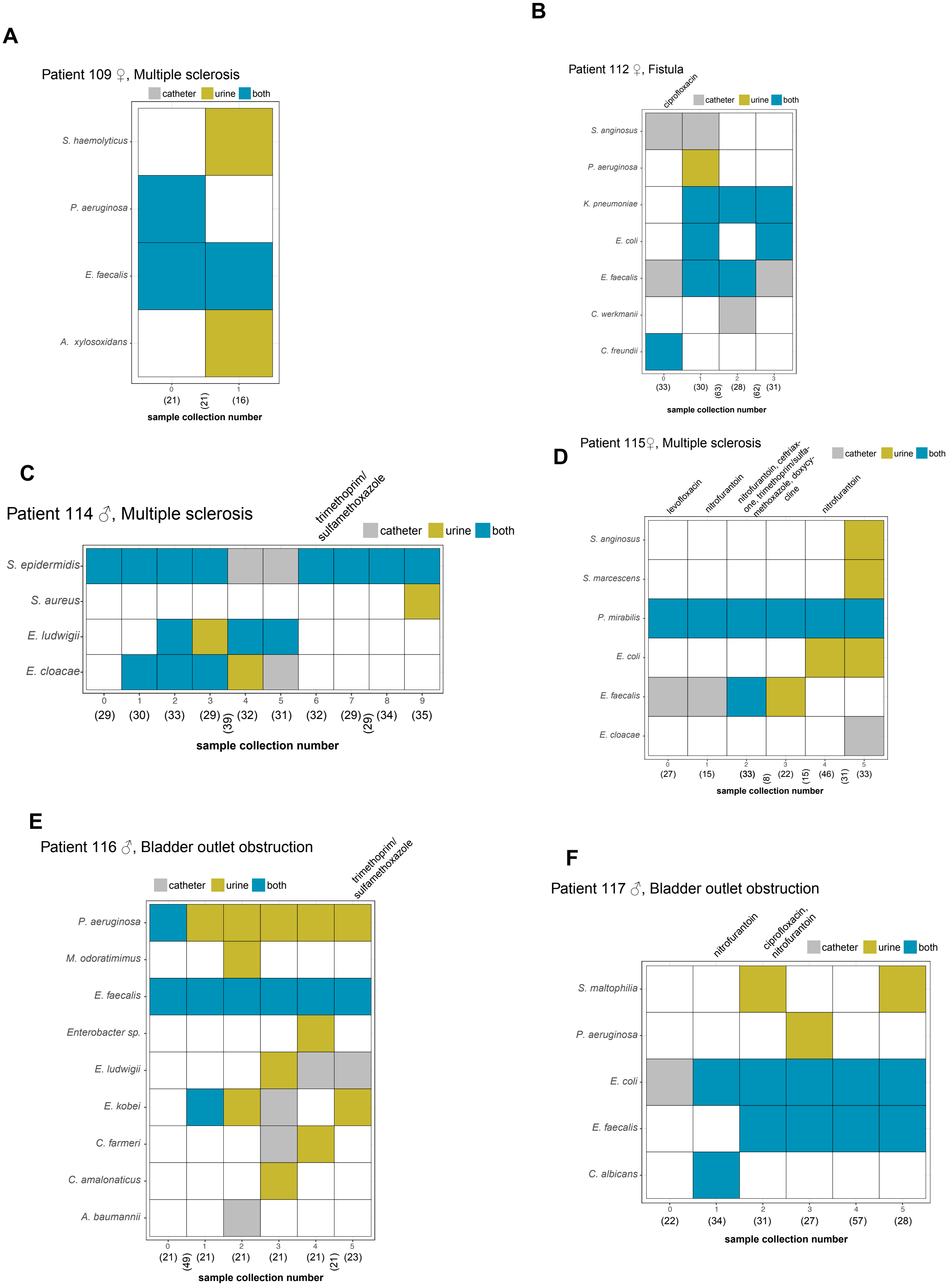

A
Patient 109 ♀, Multiple sclerosis
catheter
urine
both
S. haemolyticus
P. aeruginosa
E. faecalis
A. xylosoxidans
0
1
(21)
(21)
(16)
sample collection number
B
Patient 112 ♀, Fistula
ciprofloxacin
catheter
urine
both
S. anginosus
P. aeruginosa
K. pneumoniae
E. coli
E. faecalis
C. werkmanii
C. freundii
0
1
2
3
(33)
(30)
(63)
(28)
(62)
(31)
sample collection number
C
Patient 114 ♂, Multiple sclerosis
trimethoprim/
sulfamethoxazole
catheter
urine
both
S. epidermidis
S. aureus
E. ludwigii
E. cloacae
0
1
2
3
4
5
6
7
8
9
(29)
(30)
(33)
(29)
(39)
(32)
(31)
(32)
(29)
(29)
(34)
(35)
sample collection number
D
Patient 115♀, Multiple sclerosis
levofloxacin
nitrofurantoin
nitrofurantoin, ceftriax-
one, trimethoprim/sulfa-
methoxazole, doxycy-
cline
nitrofurantoin
catheter
urine
both
S. anginosus
S. marcescens
P. mirabilis
E. coli
E. faecalis
E. cloacae
0
1
2
3
4
5
(27)
(15)
(33)
(8)
(22)
(15)
(46)
(31)
(33)
sample collection number
E
Patient 116 ♂, Bladder outlet obstruction
trimethoprim/
sulfamethoxazole
catheter
urine
both
P. aeruginosa
M. odoratimimus
E. faecalis
Enterobacter sp.
E. ludwigii
E. kobei
C. farmeri
C. amalonaticus
A. baumannii
0
1
2
3
4
5
(21)
(49)
(21)
(21)
(21)
(21)
(21)
(23)
sample collection number
F
Patient 117 ♂, Bladder outlet obstruction
nitrofurantoin
ciprofloxacin,
nitrofurantoin
catheter
urine
both
S. maltophilia
P. aeruginosa
E. coli
E. faecalis
C. albicans
0
1
2
3
4
5
(22)
(34)
(31)
(27)
(57)
(28)
sample collection number

Supplementary Figure 4. Species identification, catheter dwell times, and antibiotics prescribed to long-term catheterized patients. Sample collection information for Patients 109, 112, 114, 115, 116, and 117 (**A-F).** Collection period is indicated on the x-axis with the duration of the period in parenthesis (days). The duration (days) of missed collection periods due to hospitalization or other causes are listed in vertical parenthesis between collection periods. Species detected within the patient are indicated on the y-axis, with gray denoting the species isolated from the catheter, yellow from the urine, and blue from the catheter and the urine at the given collection point. The antibiotics prescribed over the collection period are indicated at the top of each column. Patients with greater than one collection with at least two different species identified are included.

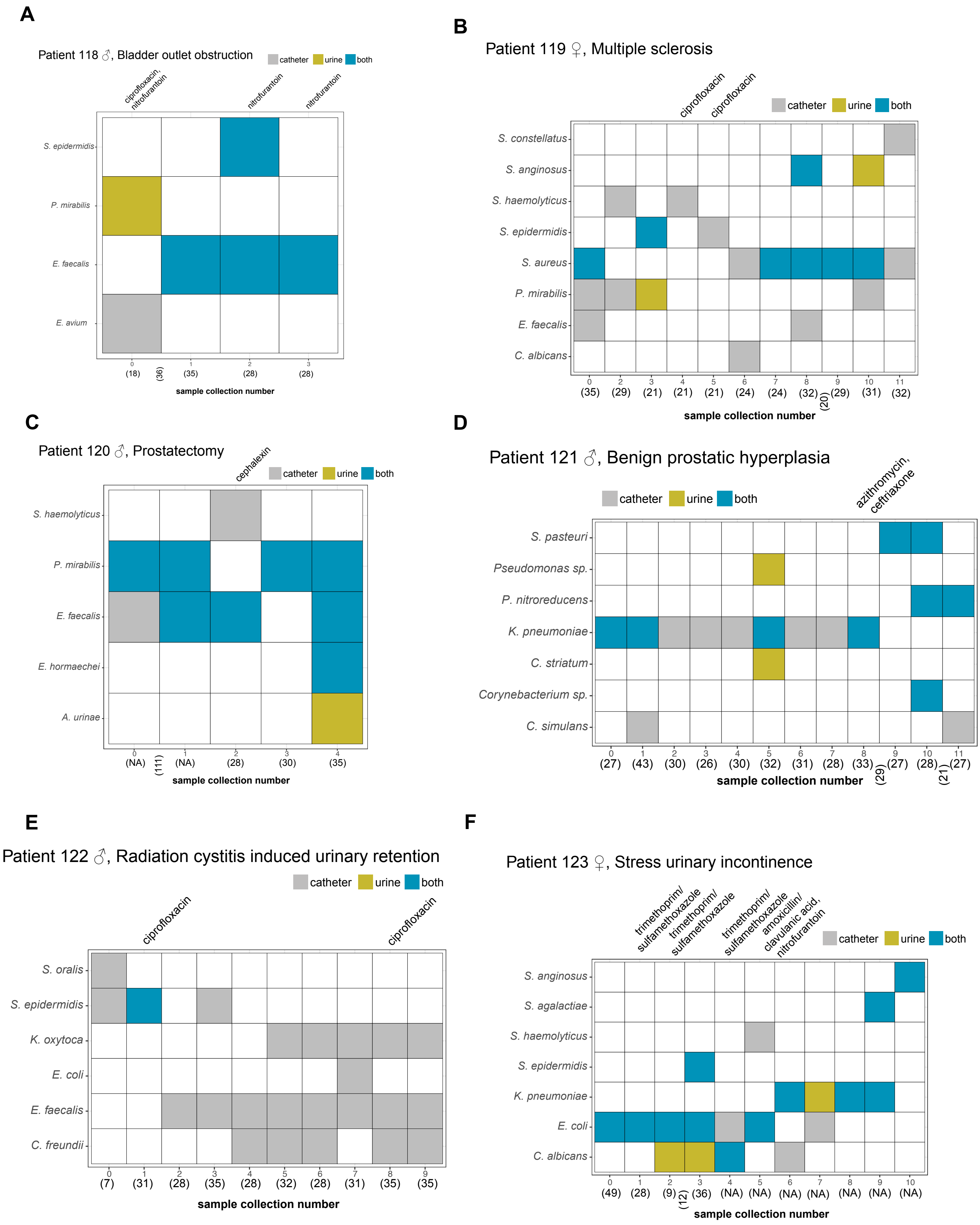
A
Patient 118 ♂, Bladder outlet obstruction
catheter urine both
ciprofloxacin, nitrofurantoin
nitrofurantoin
nitrofurantoin
S. epidermidis
P. mirabilis
E. faecalis
E. avium
0 (18)
(36)
1 (35)
2 (28)
3 (28)
sample collection number
B
Patient 119 ♀, Multiple sclerosis
ciprofloxacin
ciprofloxacin
catheter urine both
S. constellatus
S. anginosus
S. haemolyticus
S. epidermidis
S. aureus
P. mirabilis
E. faecalis
C. albicans
0 (35)
2 (29)
3 (21)
4 (21)
5 (21)
6 (24)
7 (24)
8 (32)
(20)
9 (29)
10 (31)
11 (32)
sample collection number
C
Patient 120 ♂, Prostatectomy
cephalexin
catheter urine both
S. haemolyticus
P. mirabilis
E. faecalis
E. hormaechei
A. urinae
0 (NA)
(111)
1 (NA)
2 (28)
3 (30)
4 (35)
sample collection number
D
Patient 121 ♂, Benign prostatic hyperplasia
azithromycin, ceftriaxone
catheter urine both
S. pasteuri
Pseudomonas sp.
P. nitroreducens
K. pneumoniae
C. striatum
Corynebacterium sp.
C. simulans
0 (27)
1 (43)
2 (30)
3 (26)
4 (30)
5 (32)
6 (31)
7 (28)
8 (33)
(29)
9 (27)
10 (28)
(21)
11 (27)
sample collection number
E
Patient 122 ♂, Radiation cystitis induced urinary retention
catheter urine both
ciprofloxacin
ciprofloxacin
S. oralis
S. epidermidis
K. oxytoca
E. coli
E. faecalis
C. freundii
0 (7)
1 (31)
2 (28)
3 (35)
4 (28)
5 (32)
6 (28)
7 (31)
8 (35)
9 (35)
sample collection number
F
Patient 123 ♀, Stress urinary incontinence
trimethoprim/ sulfamethoxazole
trimethoprim/ sulfamethoxazole
trimethoprim/ sulfamethoxazole
amoxicillin/ clavulanic acid, nitrofurantoin
catheter urine both
S. anginosus
S. agalactiae
S. haemolyticus
S. epidermidis
K. pneumoniae
E. coli
C. albicans
0 (49)
1 (28)
2 (9)
(12)
3 (36)
4 (NA)
5 (NA)
6 (NA)
7 (NA)
8 (NA)
9 (NA)
10 (NA)
sample collection number

Supplementary Figure 5. Species identification, catheter dwell times, and antibiotics prescribed to long-term catheterized patients. Sample collection information for Patients 118, 119, 120, 121, 122, and 123 (**A-F).** Collection period is indicated on the x-axis with the duration of the period in parenthesis (days). The duration (days) of missed collection periods due to hospitalization or other causes are listed in vertical parenthesis between collection periods. Species detected within the patient are indicated on the y-axis, with gray denoting the species isolated from the catheter, yellow from the urine, and blue from the catheter and the urine at the given collection point. The antibiotics prescribed over the collection period are indicated at the top of each column. Patients with greater than one collection with at least two different species identified are included.

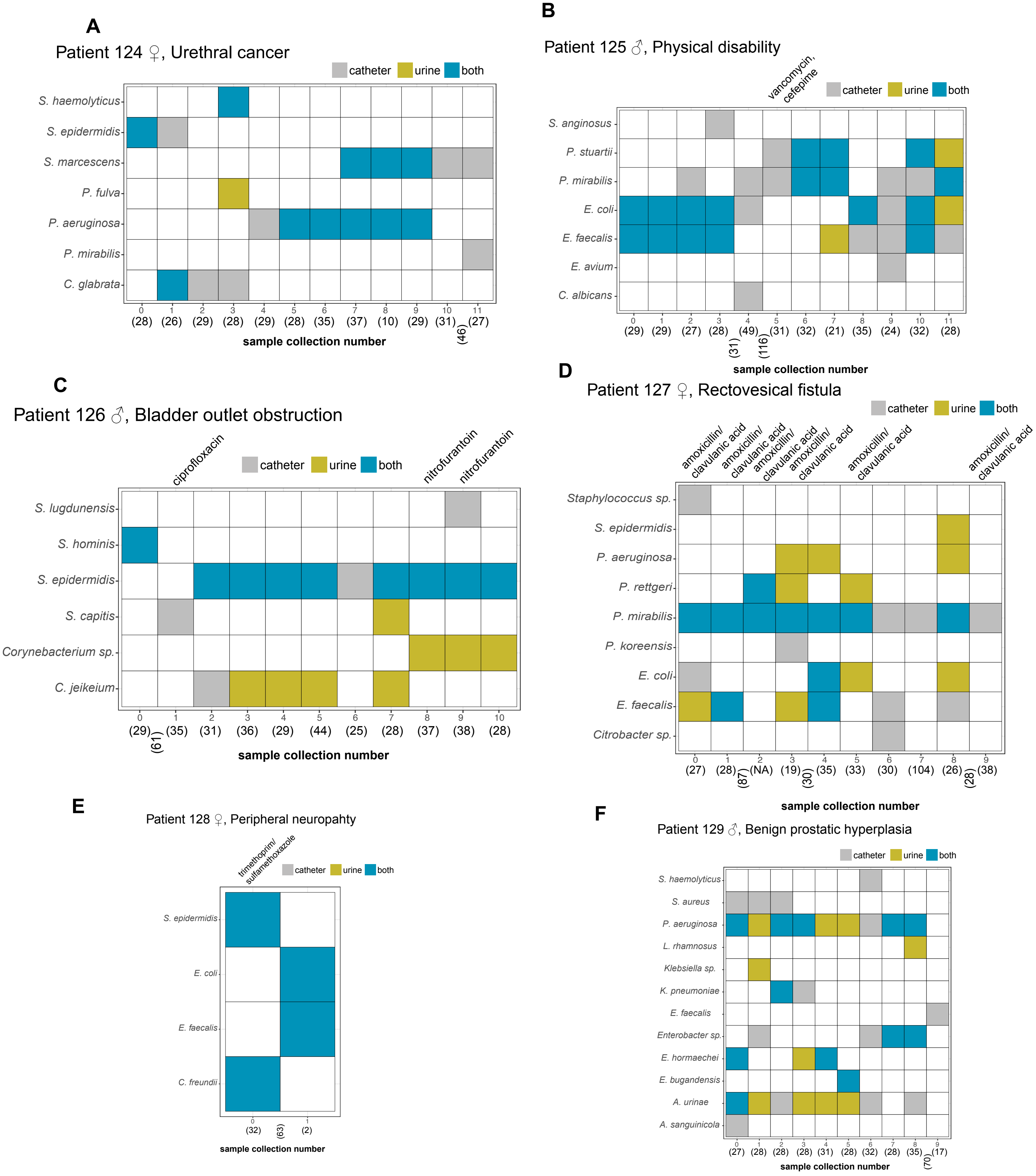

A
Patient 124 ♀, Urethral cancer
catheter
urine
both
S. haemolyticus
S. epidermidis
S. marcescens
P. fulva
P. aeruginosa
P. mirabilis
C. glabrata
sample collection number
B
Patient 125 ♂, Physical disability
vancomycin, cefepime
S. anginosus
P. stuartii
P. mirabilis
E. coli
E. faecalis
E. avium
C. albicans
C
Patient 126 ♂, Bladder outlet obstruction
ciprofloxacin
nitrofurantoin
nitrofurantoin
S. lugdunensis
S. hominis
S. epidermidis
S. capitis
Corynebacterium sp.
C. jeikeium
D
Patient 127 ♀, Rectovesical fistula
amoxicillin/ clavulanic acid
Staphylococcus sp.
S. epidermidis
P. aeruginosa
P. rettgeri
P. mirabilis
P. koreensis
E. coli
E. faecalis
Citrobacter sp.
E
Patient 128 ♀, Peripheral neuropahty
trimethoprim/ sulfamethoxazole
S. epidermidis
E. coli
E. faecalis
C. freundii
F
Patient 129 ♂, Benign prostatic hyperplasia
S. haemolyticus
S. aureus
P. aeruginosa
L. rhamnosus
Klebsiella sp.
K. pneumoniae
E. faecalis
Enterobacter sp.
E. hormaechei
E. bugandensis
A. urinae
A. sanguinicola

Supplementary Figure 6. Species identification, catheter dwell times, and antibiotics prescribed to long-term catheterized patients. Sample collection information for Patients 124, 125, 126, 127, 128, and 129 (**A-F).** Collection period is indicated on the x-axis with the duration of the period in parenthesis (days). The duration (days) of missed collection periods due to hospitalization or other causes are listed in vertical parenthesis between collection periods. Species detected within the patient are indicated on the y-axis, with gray denoting the species isolated from the catheter, yellow from the urine, and blue from the catheter and the urine at the given collection point. The antibiotics prescribed over the collection period are indicated at the top of each column. Patients with greater than one collection with at least two different species identified are included.

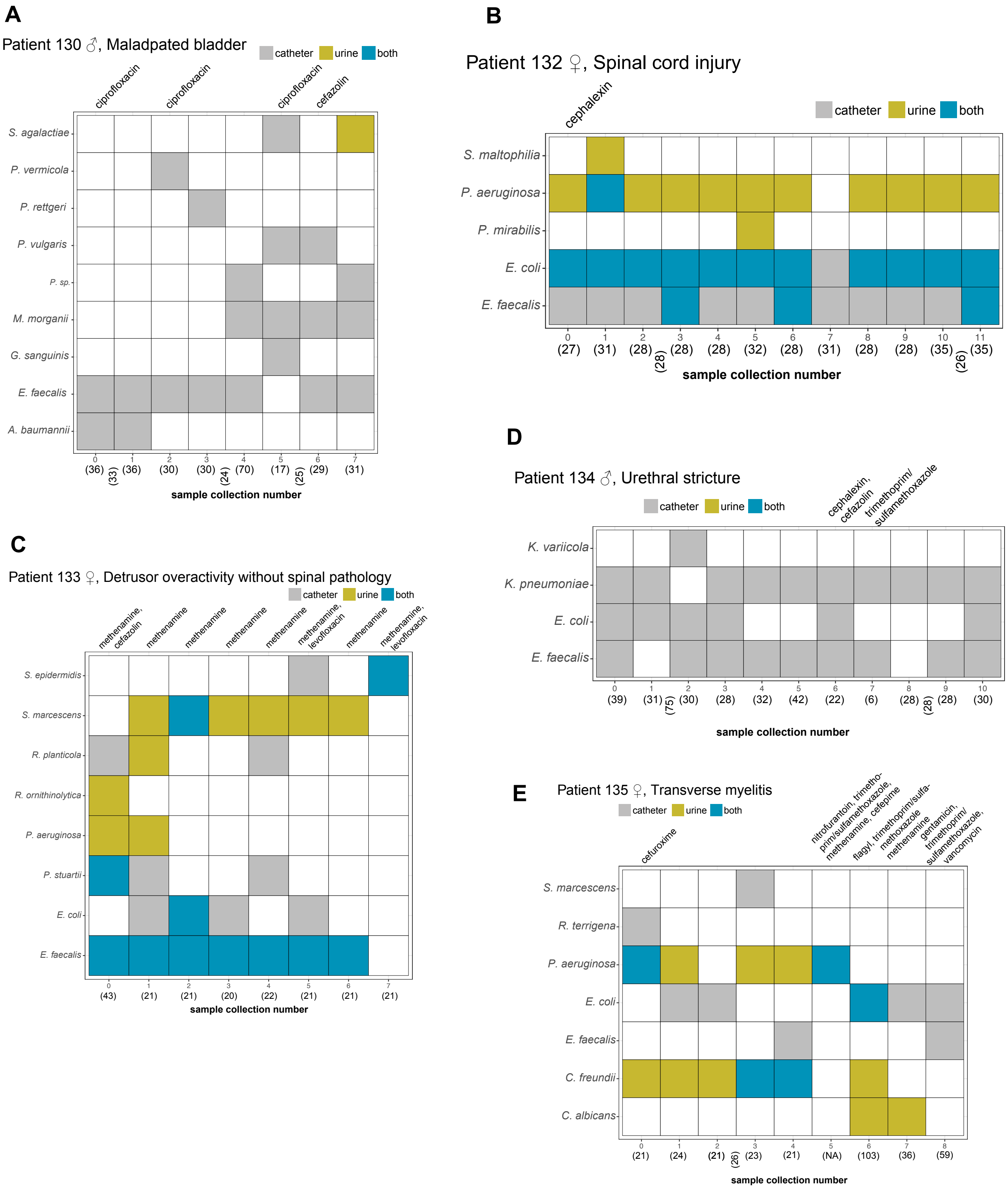
A
Patient 130 ♂, Maladpated bladder
catheter
urine
both
ciprofloxacin
ciprofloxacin
ciprofloxacin
cefazolin
S. agalactiae
P. vermicola
P. rettgeri
P. vulgaris
P. sp.
M. morganii
G. sanguinis
E. faecalis
A. baumannii
0 1 2 3 4 5 6 7
(36) (33) (36) (30) (30) (24) (70) (17) (25) (29) (31)
sample collection number
B
Patient 132 ♀, Spinal cord injury
cephalexin
catheter
urine
both
S. maltophilia
P. aeruginosa
P. mirabilis
E. coli
E. faecalis
0 1 2 3 4 5 6 7 8 9 10 11
(27) (31) (28) (28) (28) (28) (32) (28) (31) (28) (28) (35) (26) (35)
sample collection number
C
Patient 133 ♀, Detrusor overactivity without spinal pathology
catheter
urine
both
methenamine, cefazolin
methenamine
methenamine
methenamine
methenamine
methenamine, levofloxacin
methenamine
methenamine, levofloxacin
S. epidermidis
S. marcescens
R. planticola
R. ornithinolytica
P. aeruginosa
P. stuartii
E. coli
E. faecalis
0 1 2 3 4 5 6 7
(43) (21) (21) (20) (22) (21) (21) (21)
sample collection number
D
Patient 134 ♂, Urethral stricture
catheter
urine
both
cephalexin, cefazolin
trimethoprim/ sulfamethoxazole
K. variicola
K. pneumoniae
E. coli
E. faecalis
0 1 2 3 4 5 6 7 8 9 10
(39) (31) (75) (30) (28) (32) (42) (22) (6) (28) (28) (28) (30)
sample collection number
E
Patient 135 ♀, Transverse myelitis
catheter
urine
both
cefuroxime
nitrofurantoin, trimethoprim/sulfamethoxazole, methenamine, cefepime
flagyl, trimethoprim/sulfamethoxazole
methenamine
gentamicin, trimethoprim/sulfamethoxazole, vancomycin
S. marcescens
R. terrigena
P. aeruginosa
E. coli
E. faecalis
C. freundii
C. albicans
0 1 2 3 4 5 6 7 8
(21) (24) (21) (26) (23) (21) (NA) (103) (36) (59)
sample collection number

Supplementary Figure 7. Species identification, catheter dwell times, and antibiotics prescribed to long-term catheterized patients. Sample collection information for Patients 130, 132, 133, 134, and 135 (**A-E).** Collection period is indicated on the x-axis with the duration of the period in parenthesis (days). The duration (days) of missed collection periods due to hospitalization or other causes are listed in vertical parenthesis between collection periods. Species detected within the patient are indicated on the y-axis, with gray denoting the species isolated from the catheter, yellow from the urine, and blue from the catheter and the urine at the given collection point. The antibiotics prescribed over the collection period are indicated at the top of each column. Patients with greater than one collection with at least two different species identified are included.

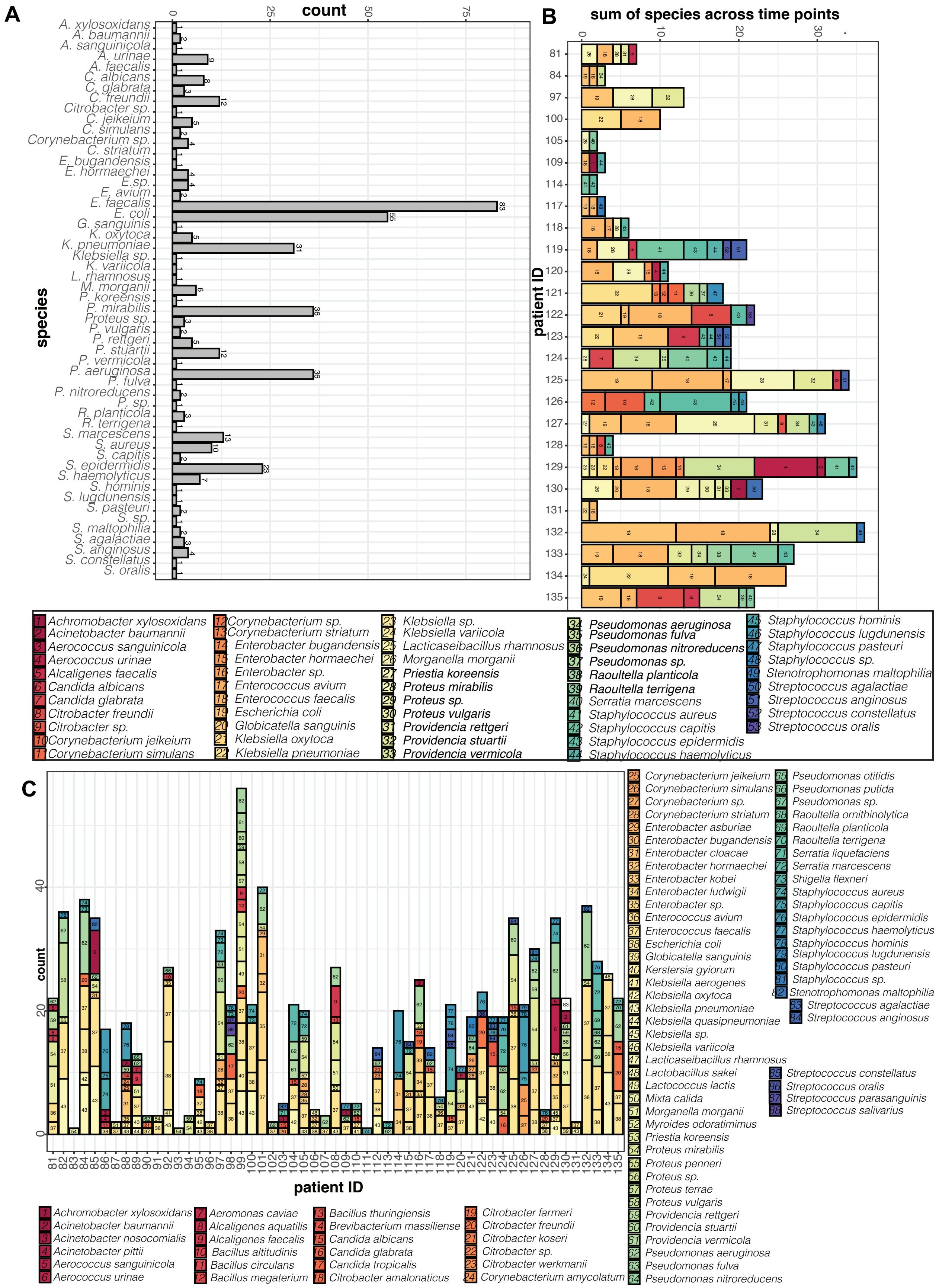
A
count
species
A. xylosoxidans
A. baumannii
A. sanguinicola
A. urinae
A. faecalis
C. albicans
C. glabrata
C. freundii
Citrobacter sp.
C. jeikeium
C. simulans
Corynebacterium sp.
C. striatum
E. bugandensis
E. hormaechei
E.sp.
E. avium
E. faecalis
E. coli
G. sanguinis
K. oxytoca
K. pneumoniae
Klebsiella sp.
K. variicola
L. rhamnosus
M. morganii
P. koreensis
P. mirabilis
Proteus sp.
P. vulgaris
P. rettgeri
P. stuartii
P. vermicola
P. aeruginosa
P. fulva
P. nitroreducens
P. sp.
R. planticola
R. terrigena
S. marcescens
S. aureus
S. capitis
S. epidermidis
S. haemolyticus
S. hominis
S. lugdunensis
S. pasteuri
S. sp.
S. maltophilia
S. agalactiae
S. anginosus
S. constellatus
S. oralis
B
sum of species across time points
patient ID
1 Achromobacter xylosoxidans
2 Acinetobacter baumannii
3 Aerococcus sanguinicola
4 Aerococcus urinae
5 Alcaligenes faecalis
6 Candida albicans
7 Candida glabrata
8 Citrobacter freundii
9 Citrobacter sp.
10 Corynebacterium jeikeium
11 Corynebacterium simulans
12 Corynebacterium sp.
13 Corynebacterium striatum
14 Enterobacter bugandensis
15 Enterobacter hormaechei
16 Enterobacter sp.
17 Enterococcus avium
18 Enterococcus faecalis
19 Escherichia coli
20 Globicatella sanguinis
21 Klebsiella oxytoca
22 Klebsiella pneumoniae
23 Klebsiella sp.
24 Klebsiella variicola
25 Lacticaseibacillus rhamnosus
26 Morganella morganii
27 Priestia koreensis
28 Proteus mirabilis
29 Proteus sp.
30 Proteus vulgaris
31 Providencia rettgeri
32 Providencia stuartii
33 Providencia vermicola
34 Pseudomonas aeruginosa
35 Pseudomonas fulva
36 Pseudomonas nitroreducens
37 Pseudomonas sp.
38 Raoultella planticola
39 Raoultella terrigena
40 Serratia marcescens
41 Staphylococcus aureus
42 Staphylococcus capitis
43 Staphylococcus epidermidis
44 Staphylococcus haemolyticus
45 Staphylococcus hominis
46 Staphylococcus lugdunensis
47 Staphylococcus pasteuri
48 Staphylococcus sp.
49 Stenotrophomonas maltophilia
50 Streptococcus agalactiae
51 Streptococcus anginosus
52 Streptococcus constellatus
53 Streptococcus oralis
C
count
patient ID
1 Achromobacter xylosoxidans
2 Acinetobacter baumannii
3 Acinetobacter nosocomialis
4 Acinetobacter pittii
5 Aerococcus sanguinicola
6 Aerococcus urinae
7 Aeromonas caviae
8 Alcaligenes aquatilis
9 Alcaligenes faecalis
10 Bacillus altitudinis
11 Bacillus circulans
12 Bacillus megaterium
13 Bacillus thuringiensis
14 Brevibacterium massiliense
15 Candida albicans
16 Candida glabrata
17 Candida tropicalis
18 Citrobacter amalonaticus
19 Citrobacter farmeri
20 Citrobacter freundii
21 Citrobacter koseri
22 Citrobacter sp.
23 Citrobacter werkmanii
24 Corynebacterium amycolatum
25 Corynebacterium jeikeium
26 Corynebacterium simulans
27 Corynebacterium sp.
28 Corynebacterium striatum
29 Enterobacter asburiae
30 Enterobacter bugandensis
31 Enterobacter cloacae
32 Enterobacter hormaechei
33 Enterobacter kobei
34 Enterobacter ludwigii
35 Enterobacter sp.
36 Enterococcus avium
37 Enterococcus faecalis
38 Escherichia coli
39 Globicatella sanguinis
40 Kerstersia gyiorum
41 Klebsiella aerogenes
42 Klebsiella oxytoca
43 Klebsiella pneumoniae
44 Klebsiella quasipneumoniae
45 Klebsiella sp.
46 Klebsiella variicola
47 Lacticaseibacillus rhamnosus
48 Lactobacillus sakei
49 Lactococcus lactis
50 Mixta calida
51 Morganella morganii
52 Myroides odoratimimus
53 Priestia koreensis
54 Proteus mirabilis
55 Proteus penneri
56 Proteus sp.
57 Proteus terrae
58 Proteus vulgaris
59 Providencia rettgeri
60 Providencia stuartii
61 Providencia vermicola
62 Pseudomonas aeruginosa
63 Pseudomonas fulva
64 Pseudomonas nitroreducens
65 Pseudomonas otitidis
66 Pseudomonas putida
67 Pseudomonas sp.
68 Raoultella ornithinolytica
69 Raoultella planticola
70 Raoultella terrigena
71 Serratia liquefaciens
72 Serratia marcescens
73 Shigella flexneri
74 Staphylococcus aureus
75 Staphylococcus capitis
76 Staphylococcus epidermidis
77 Staphylococcus haemolyticus
78 Staphylococcus hominis
79 Staphylococcus lugdunensis
80 Staphylococcus pasteuri
81 Staphylococcus sp.
82 Stenotrophomonas maltophilia
83 Streptococcus agalactiae
84 Streptococcus anginosus
85 Streptococcus constellatus
86 Streptococcus oralis
87 Streptococcus parasanguinis
88 Streptococcus salivarius

Supplementary Figure 8. Species composition of the polymicrobial communities of long-term catheterized patients with raw sequencing data. A) Number of occurrences of indicated species per patient per collection period detected in the catheter and/or urine samples in the long-term catheterization cohort for which sequencing data has been deposited at BioProject ID PRJNA956906. Numbers above bars correspond to number of occurrences in dataset. The overall distribution of species detected is to be compared to **Fig 3A** in the main text. **B)** Detected species, indicated by colored key and corresponding numbers, by patient over all collection periods for which raw sequencing data are available (BioProject ID PRJNA956906). Raw sequencing files were recovered for samples collected after 1/14/2020. **C)** Detected species, indicated by colored key and corresponding numbers, by patient over all collection periods.

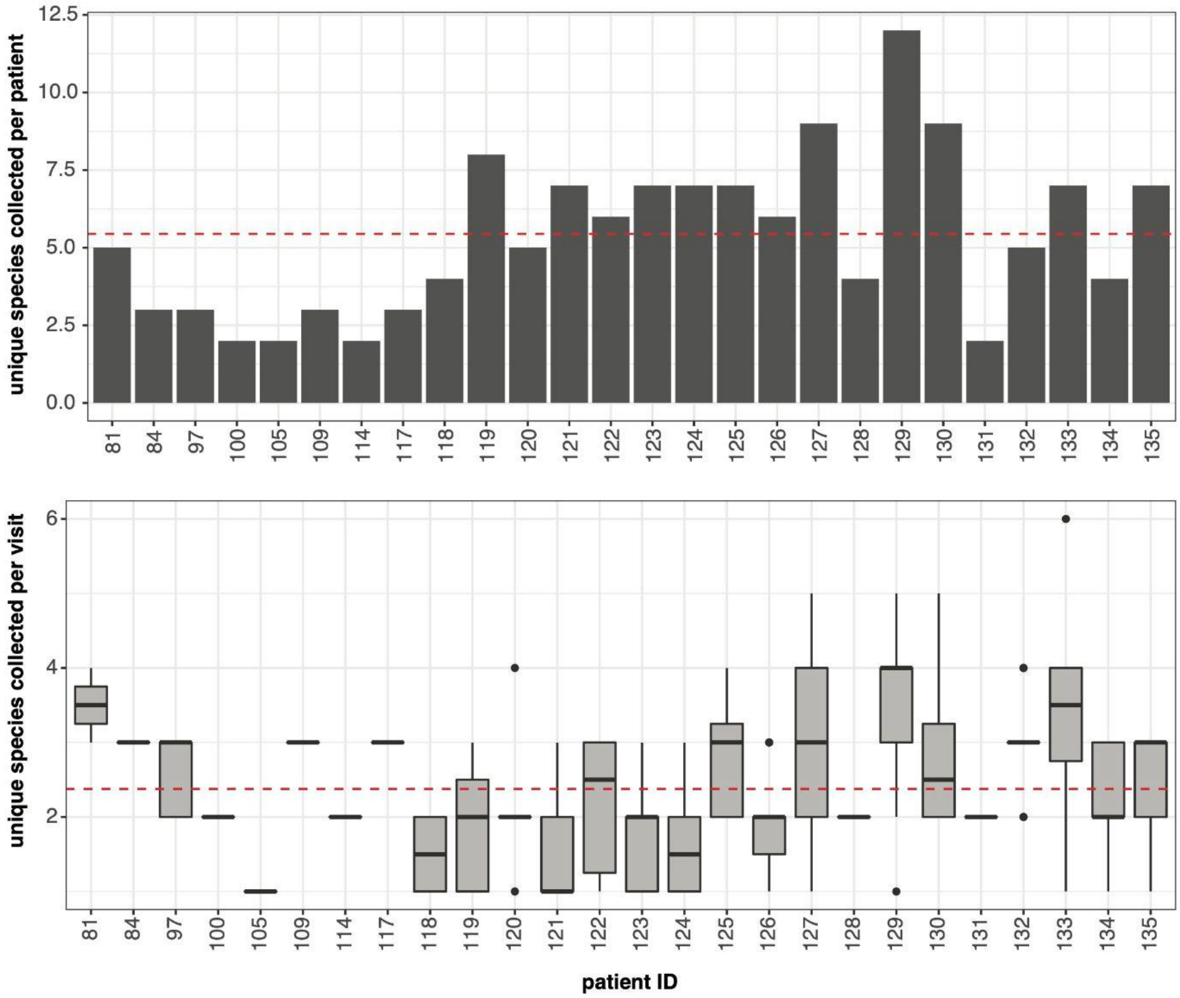

Supplementary Figure 9. The polymicrobial community composition of long-term catheterized patients averages 5-6 species per patient but only 2-3 species per collection. The number of unique species identified per patient **(top)** for which raw sequencing data are available (BioProject ID PRJNA956906). Boxplots of the number of unique species identified per patient per visit for which raw sequencing data are available (BioProject ID PRJNA956906) **(bottom)**. Average is indicated by a dashed red line.

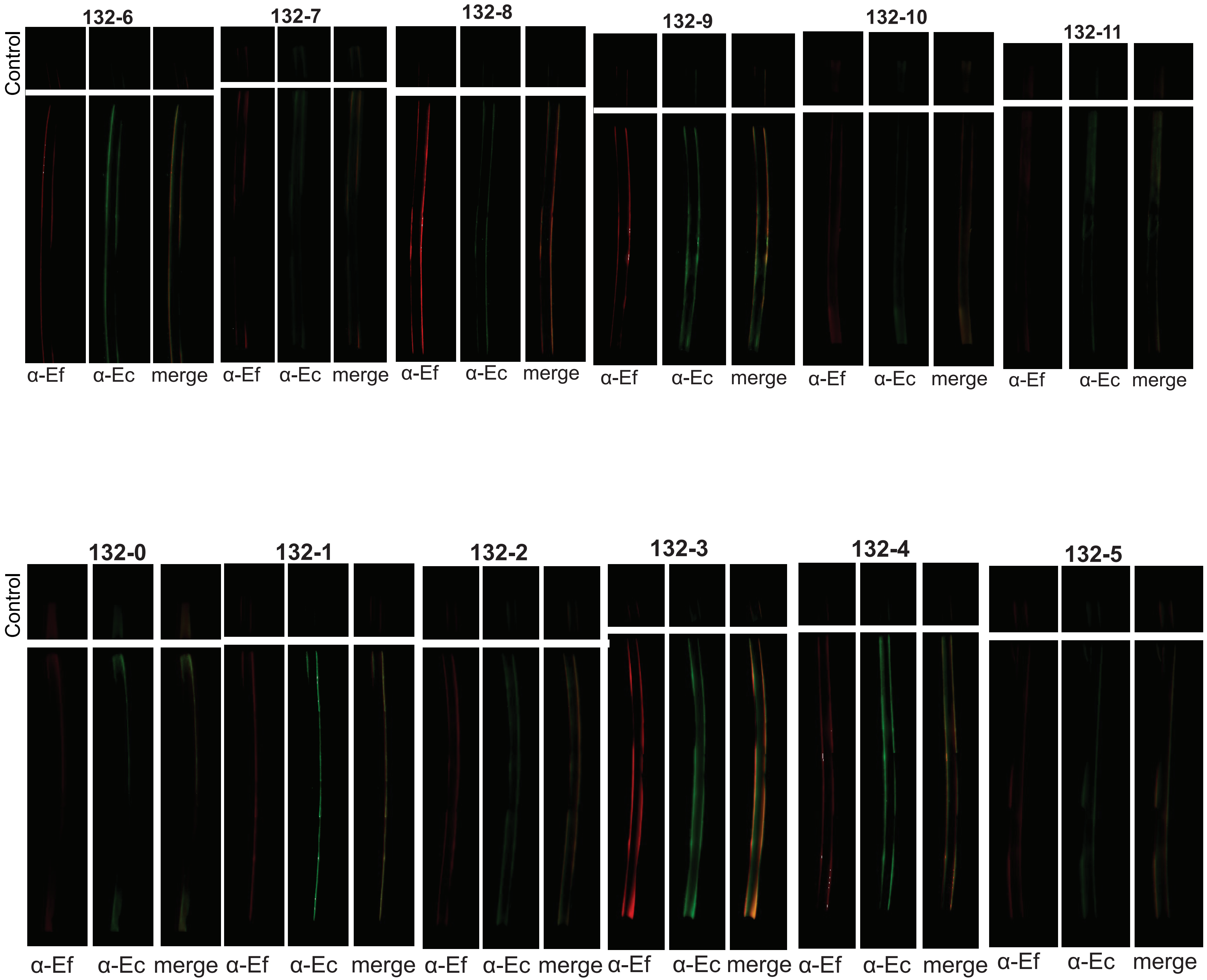

132-6
132-7
132-8
132-9
132-10
132-11
Control
α-Ef α-Ec merge α-Ef α-Ec merge α-Ef α-Ec merge α-Ef α-Ec merge α-Ef α-Ec merge α-Ef α-Ec merge
132-0
132-1
132-2
132-3
132-4
132-5
Control
α-Ef α-Ec merge α-Ef α-Ec merge α-Ef α-Ec merge α-Ef α-Ec merge α-Ef α-Ec merge α-Ef α-Ec merge

Supplementary Figure 10. *E. faecalis* and *E. coli* co-localize on catheters isolated from Patient 132. Immunohistochemistry staining of formalin fixed catheter portion incubated with the indicated primary antibodies for Patient 132.

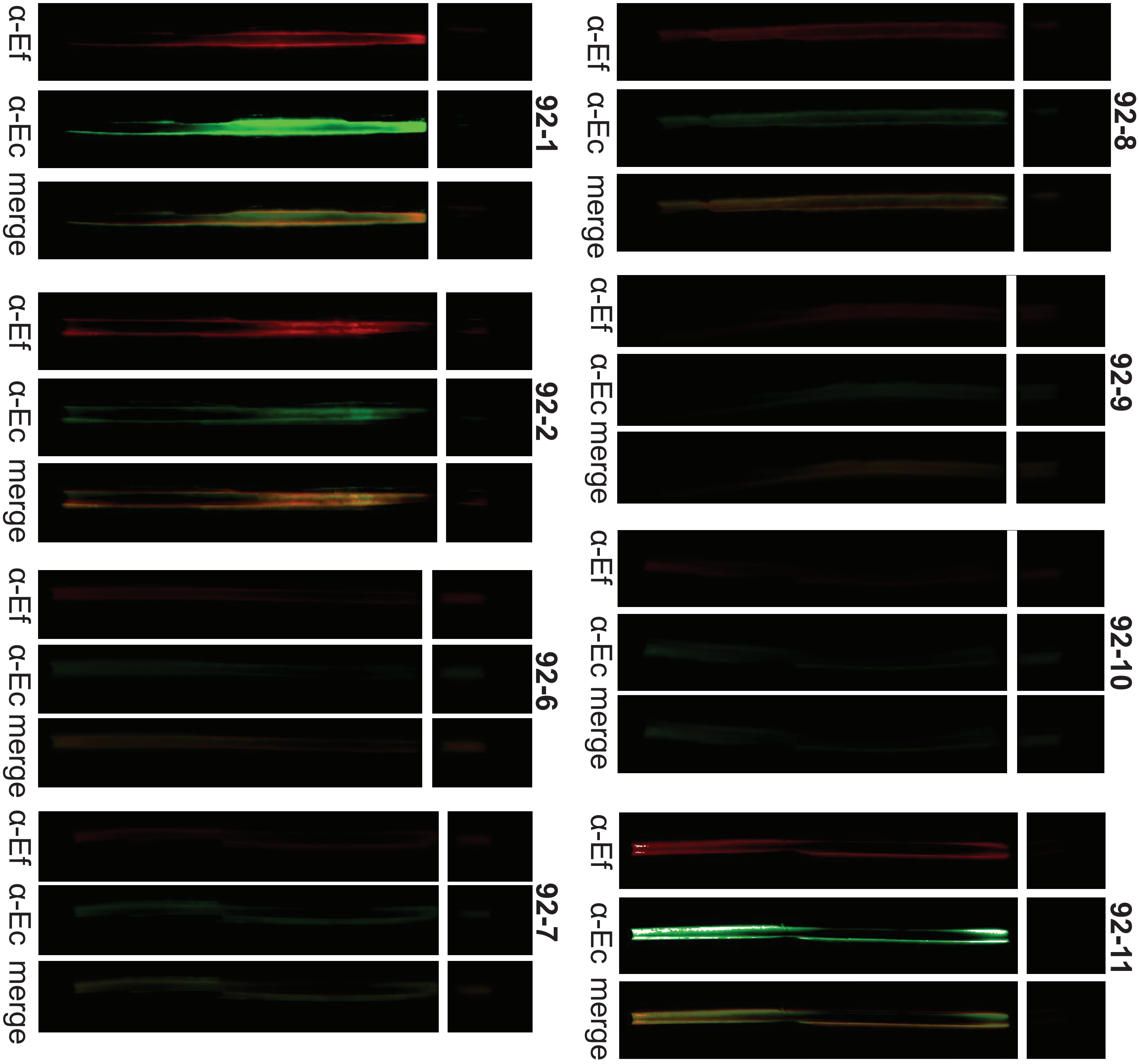

Supplementary Figure 11. ***E. faecalis*** **and** ***E. coli*** **co-localize on catheters isolated from Patient 92.** Immunohistochemistry staining of formalin fixed catheter portion incubated with the indicated primary antibodies for Patient 92.

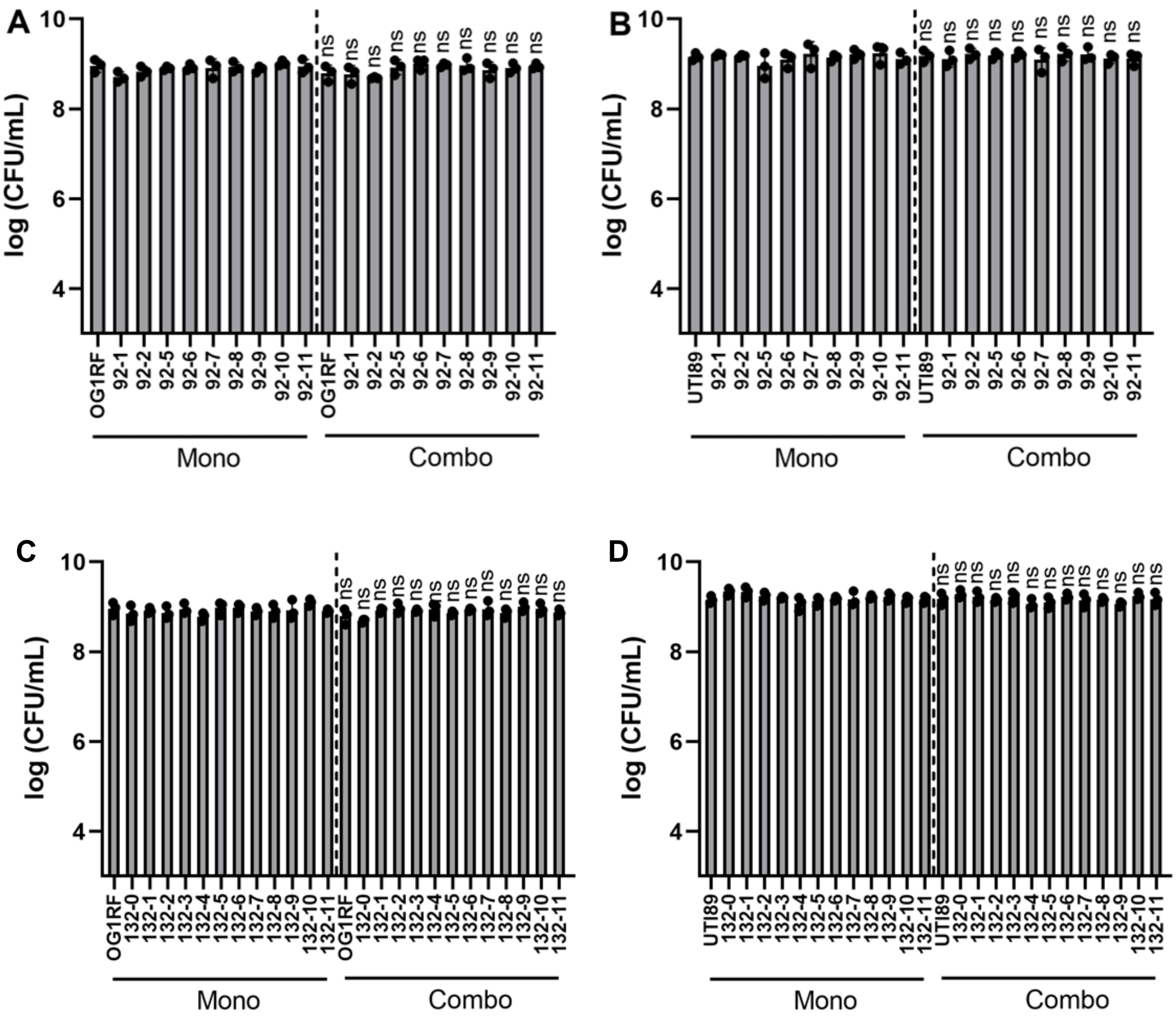

Supplementary Figure 12. ***E. coli*** **clinical isolates do not promote** ***E. faecalis*** **growth from Patient 92 and Patient 132 in rich BHI media. (A ~ D)** Growths (log CFU/mL) of clinical *E. faecalis* **(A, C)** or *E. coli* **(B, D)** isolates from Patient 92 and Patient 132, respectively, in monocultures (Mono) or mixed cultures (Combo) in BHI. Each pair of *E.* faecalis and *E. coli* isolates were collected at the same collection period from Patients 92 and 132. Growth of prototypical strains *E.faecalis* OG1RF with *E. coli* UTI89 are included. Comparisons conducted using unpaired t test. *P ≤ 0.05, **P <

0.01, ***P < 0.001, ****P < 0.0001, ns indicates not significant. Error bars indicate standard deviation.

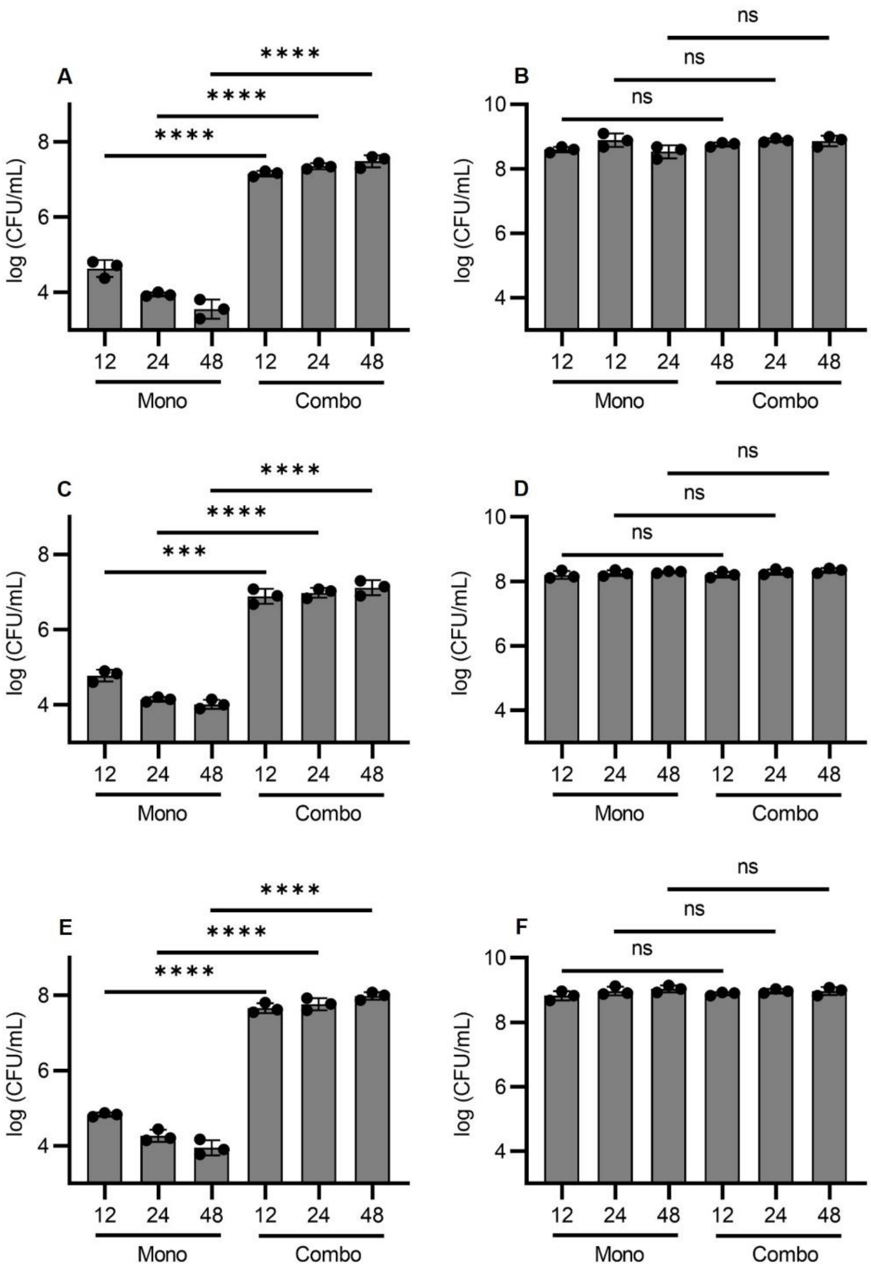

Supplementary Figure 13. ***E. coli*** **isolates significantly promote** ***E. faecalis*** **isolates growth in AUM minimal media across 12, 24, and 48-hour time points**. **(A & B)** Growths (log CFU/mL) of *E. faecalis* OG1RF (A) or *E. coli* UTI89 (B) isolates at 12 hours, 24 hours, and 48 hours in monocultures (Mono) or mixed cultures (Combo) in AUM. **(C & D)** Growths (log CFU/mL) of *E. faecalis* 92-1 (C) or *E. col*i 92-1 (D) isolates at 12 hours, 24 hours, and 48 hours

in monocultures (Mono) or mixed cultures (Combo) in AUM. *E. faecalis* 92-1 and *E. coli* 92-1 isolates were collected at the collection 1 from Patient 92. **(E & F)** Growths (log CFU/mL) of *E. faecalis* 132-0 **(E)** or *E. coli* 132-0 **(F)** isolates at 12 hours, 24 hours, and 48 hours in monocultures (Mono) or mixed cultures (Combo) in AUM. *E. faecalis* 132-0 and *E. coli* 132-0 isolates were collected at the collection 0 from Patient 132. Comparisons conducted using unpaired t test. *P ≤ 0.05, **P < 0.01, ***P < 0.001, ****P < 0.0001, ns indicates not significant. Error bars indicate standard deviation.

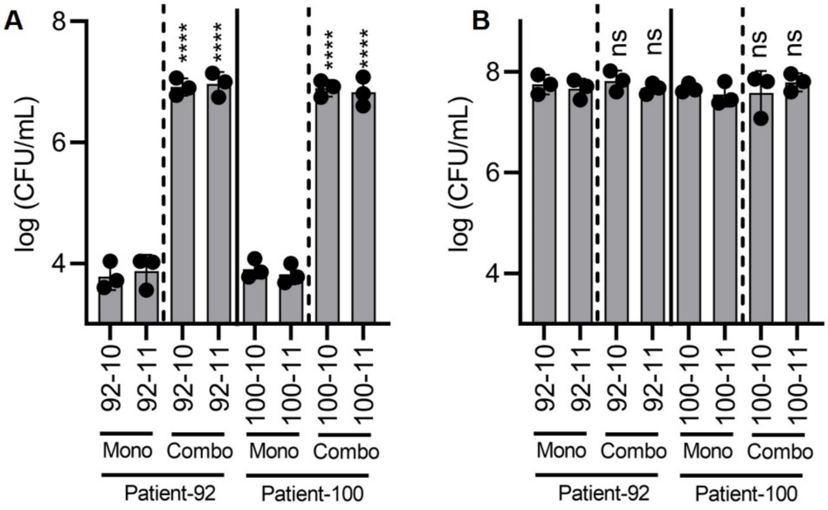

Supplementary Figure 14. Bacterial interaction between *E. faecalis* and *K. pneumoniae* isolates from Patient 92 and Patient 100 in AUM minimal media. (**A & B**) Growths (log CFU/mL) of clinical *E. faecalis* **(A)** and *K. pneumoniae* **(B)** isolates from Patients 92 and 100 in monocultures (Mono) or mixed cultures (Combo) in AUM. Each pair of *E.* faecalis and *K. pneumoniae* isolates were collected at the same collection period from Patients 92 and 100. Comparisons are conducted using unpaired t test. *P ≤ 0.05, **P < 0.01, ***P < 0.001, ****P < 0.0001, ns indicates not significant. Error bars indicate standard deviation.

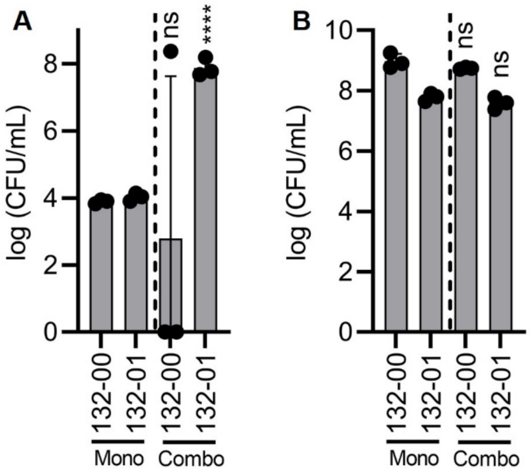

Supplementary Figure 15. Bacterial interaction between *E. faecalis* and *P. aeruginosa* isolates from Patient 132 in AUM minimal media. (**A & B**) Growths (log CFU/mL) of clinical *E. faecalis* **(A)** and *P. aeruginosa* **(B)** isolates from Patient 132 in monocultures (Mono) or mixed cultures (Combo) in AUM. Each pair of *E. faecalis* and *P. aeruginosa* isolates were collected at the same collection episode from Patient 132. Comparisons are conducted using unpaired t test. *P ≤ 0.05, **P < 0.01, ***P < 0.001, ****P < 0.0001, ns indicates not significant. Error bars indicate standard deviation.

Table S1. Single species collection periods.

site	patient	source	species
100-02	100	catheter	*Escherichia coli*
100-03	100	catheter	*Escherichia coli*
100-04	100	catheter	*Enterococcus faecalis*
100-05	100	catheter	*Klebsiella pneumoniae*
105-06	105	both	*Proteus mirabilis*
111-01	111	both	*Staphylococcus aureus*
114-0	114	both	*Staphylococcus epidermidis*
114-06	114	both	*Staphylococcus epidermidis*
114-07	114	both	*Staphylococcus epidermidis*
114-08	114	both	*Staphylococcus epidermidis*
117-0	117	catheter	*Escherichia coli*
118-01	118	both	*Enterococcus faecalis*
118-03	118	both	*Enterococcus faecalis*
119-04	119	catheter	*Staphylococcus haemolyticus*
119-05	119	catheter	*Staphylococcus epidermidis*
119-07	119	both	*Staphylococcus aureus*
119-09	119	both	*Staphylococcus aureus*
120-03	120	both	*Proteus mirabilis*
121-0	121	both	*Klebsiella pneumoniae*
121-02	121	catheter	*Klebsiella pneumoniae*
121-03	121	catheter	*Klebsiella pneumoniae*
121-04	121	catheter	*Klebsiella pneumoniae*
121-06	121	catheter	*Klebsiella pneumoniae*
121-07	121	catheter	*Klebsiella pneumoniae*
121-08	121	both	*Klebsiella pneumoniae*
121-09	121	both	*Staphylococcus pasteuri*
122-01	122	both	*Staphylococcus epidermidis*
122-02	122	catheter	*Enterococcus faecalis*
123-0	123	both	*Escherichia coli*
123-01	123	both	*Escherichia coli*
123-08	123	both	*Klebsiella pneumoniae*
123-10	123	both	*Streptococcus anginosus*
124-0	124	both	*Staphylococcus epidermidis*
124-02	124	both	*Candida glabrata*
124-04	124	both	*Pseudomonas aeruginosa*
124-05	124	catheter	*Pseudomonas aeruginosa*
124-06	124	both	*Pseudomonas aeruginosa*
124-10	124	catheter	*Serratia marcescens*
126-01	126	both	*Staphylococcus hominis*
126-02	126	catheter	*Staphylococcus capitis*
126-07	126	catheter	*Staphylococcus epidermidis*
127-07	127	catheter	*Proteus mirabilis*

127-09	127	catheter	*Proteus mirabilis*
129-09	129	catheter	*Enterococcus faecalis*
133-07	133	both	*Staphylococcus epidermidis*
134-08	134	catheter	*Klebsiella pneumoniae*
135-05	135	both	*Pseudomonas aeruginosa*
82-0	82	both	*Proteus vulgaris*
83-0	83	both	*Proteus mirabilis*
84-0	84	both	*Escherichia coli*
86-01	86	catheter	*Staphylococcus epidermidis*
86-05	86	both	*Staphylococcus epidermidis*
86-06	86	both	*Staphylococcus epidermidis*
86-07	86	both	*Staphylococcus aureus*
86-08	86	catheter	*Staphylococcus aureus*
88-02	88	catheter	*Staphylococcus epidermidis*
88-05	88	catheter	*Enterobacter cloacae*
88-06	88	catheter	*Klebsiella pneumoniae*
88-07	88	catheter	*Enterococcus faecalis*
88-08	88	catheter	*Staphylococcus epidermidis*
88-09	88	catheter	*Staphylococcus epidermidis*
88-10	88	catheter	*Staphylococcus epidermidis*
88-11	88	catheter	*Staphylococcus epidermidis*
91-0	91	both	*Escherichia coli*
92-0	92	both	*Klebsiella aerogenes*
92-03	92	both	*Escherichia coli*
92-04	92	both	*Escherichia coli*
93-0	93	both	*Proteus mirabilis*
94-0	94	both	*Proteus mirabilis*
97-03	97	both	*Serratia marcescens*

*****Source indicates where the species was isolated from the catheter sample, urine sample, or both for the indicated patient and collection period.

Table S2. Significant co-occurrences from collection period 0.

Genus 1	Genus 2	g1_inc	g2_inc	obs	exp	p_lt	p_gt
Alcaligenes	Bacillus	1	1	1	0	1	0.01887
Candida	Achromobacter	2	1	1	0	1	0.03774
Candida	Serratia	2	5	2	0.2	1	0.00726
Candida	Stenotrophomonas	2	1	1	0	1	0.03774
Proteus	Pseudomonas	12	13	0	2.9	0.02094	1
Achromobacter	Corynebacterium	1	2	1	0	1	0.03774
Achromobacter	Stenotrophomonas	1	1	1	0	1	0.01887
Raoultella	Providencia	2	1	1	0	1	0.03774
Enterococcus	Staphylococcus	30	11	2	6.2	0.00513	0.99953
Corynebacterium	Brevibacterium	2	1	1	0	1	0.03774
Corynebacterium	Stenotrophomonas	2	1	1	0	1	0.03774

Table S3. Significant co-occurrences from collection period 1.

Genus 1	Genus 2	g1_inc	g2_inc	obs	exp	p_lt	p_gt
Klebsiella	Pseudomonas	10	10	5	2.4	0.99445	0.04004
Raoultella	Serratia	1	2	1	0	1	0.04762
Enterococcus	Staphylococcus	26	10	3	6.2	0.02326	0.99695
Morganella	Alcaligenes	3	4	3	0.3	1	0.00035
Staphylococcus	Proteus	10	10	0	2.4	0.04384	1
Serratia	Providencia	2	4	2	0.2	1	0.00697
Proteus	Pseudomonas	10	10	0	2.4	0.04384	1
Pseudomonas	Providencia	10	4	3	1	0.99812	0.03618

Table S4. Significant co-occurrences from collection period 2.

Genus 1	Genus 2	g1_inc	g2_inc	obs	exp	p_lt	p_gt
Staphylococcus	Enterococcus	9	21	2	5.1	0.02158	0.99774
Pseudomonas	Klebsiella	7	9	4	1.7	0.99514	0.04495

Table S5. Significant co-occurrences from collection period 3.

Genus 1	Genus 2	g1_inc	g2_inc	obs	exp	p_lt	p_gt
Kerstersia	Lactococcus	1	1	1	0	1	0.02703
Kerstersia	Bacillus	1	1	1	0	1	0.02703
Morganella	Proteus	3	9	3	0.7	1	0.01081
Lactococcus	Bacillus	1	1	1	0	1	0.02703

*Genus 1 and Genus 2 indicate the pairwise genera included. The number of occurrences for each genus is indicated in “g1_inc” and “g2_inc.” The observed (obs) and expected (exp) co-occurrences are also indicated. The significance is based on the probability that two species co-occur at a frequency greater (p_gt) or less (p_lt) than observed co-occurrence frequency, which can be used as p-values.

Table S6. Significant co-occurrences from collection period 4.

Genus 1	Genus 2	g1_inc	g2_inc	obs	exp	p_lt	p_gt
Proteus	Morganella	9	5	4	1.3	0.99955	0.01177
Lactococcus	Alcaligenes	1	1	1	0	1	0.02941
Aerococcus	Enterobacter	2	7	2	0.4	1	0.03743

Table S7. Significant co-occurrences from collection period 5.

Genus 1	Genus 2	g1_inc	g2_inc	obs	exp	p_lt	p_gt
Pseudomonas	Staphylococcus	14	6	0	2.5	0.0245	1
Providencia	Proteus	3	9	3	0.8	1	0.0154

Table S8. Significant co-occurrences from collection period 6.

Genus 1	Genus 2	g1_inc	g2_inc	obs	exp	p_lt	p_gt
Enterococcus	Staphylococcus	11	6	0	2.4	0.02705	1
Kerstersia	Lactococcus	1	1	1	0	1	0.03704

Table S9. Significant co-occurrences from collection period 7.

Genus 1	Genus 2	g1_inc	g2_inc	obs	exp	p_lt	p_gt
Enterococcus	Staphylococcus	12	5	0	2.4	0.02422	1
Streptococcus	Morganella	1	1	1	0	1	0.04

Table S10. Significant co-occurrences from collection period 8.

Genus 1	Genus 2	g1_inc	g2_inc	obs	exp	p_lt	p_gt
Staphylococcus	Klebsiella	6	9	0	2.3	0.02975	1
Lacticaseibacillus	Aerococcus	1	1	1	0	1	0.04348

Table S11. Significant co-occurrences from collection period 9.

Genus 1	Genus 2	g1_inc	g2_inc	obs	exp	p_lt	p_gt
Klebsiella	Staphylococcus	8	6	0	2.2	0.04025	1
Enterococcus	Staphylococcus	11	6	0	3	0.00619	1

Table S12. Significant co-occurrences from collection period 10.

Genus 1	Genus 2	g1_inc	g2_inc	obs	exp	p_lt	p_gt
Enterococcus	Staphylococcus	10	5	0	2.8	0.00654	1
Enterococcus	Klebsiella	10	5	5	2.8	1	0.02941
Proteus	Providencia	6	3	3	1	1	0.02451

Table S13. Significant co-occurrences from collection period 11.

Genus 1	Genus 2	g1_inc	g2_inc	obs	exp	p_lt	p_gt
Proteus	Providencia	6	3	3	1.2	1	0.04396

*Genus 1 and Genus 2 indicate the pairwise genera included. The number of occurrences for each genus is indicated in "g1_inc" and "g2_inc." The observed (obs) and expected (exp) co-occurrences are also indicated. The significance is based on the probability that two species co-occur at a frequency greater (p_gt) or less (p_lt) than observed co-occurrence frequency, which can be used as p-values.

Table S14. Twenty-two pairs of *E. faecalis* and *E. coli* strains for studying bacterial interactions in polymicrobial community

Origin	*Enterococcus faecalis*	*Escherichia coli*
**Model strain**	OG1RF	UTI89
**Patient 92**	EF92-1[a]	EC92-1[b]
	EF92-2	EC92-2
	EF92-5	EC92-5
	EF92-6	EC92-6
	EF92-7	EC92-7
	EF92-8	EC92-8
	EF92-9	EC92-9
	EF92-10	EC92-10
	EF92-11	EC92-11
**Patient 132**	EF132-0	EC132-0
	EF132-1	EC132-1
	EF132-2	EC132-2
	EF132-3	EC132-3
	EF132-4	EC132-4
	EF132-5	EC132-5
	EF132-6	EC132-6
	EF132-7	EC132-7
	EF132-8	EC132-8
	EF132-9	EC132-9
	EF132-10	EC132-10
	EF132-11	EC132-11

[a]EF represents the *E. faecalis* species. EF92-1 represents the *E. faecalis* isolate collected from the second clinical visit of Patient 92.

[b]EC represents the *E. coli* species. EC92-1 represents the *E. coli* isolate collected from the second clinical visit of Patient 92.

Table S15. Four pairs of *E. faecalis* and *K. pneumoniae* strains from Patients 92 and 100 for studying bacterial interactions in polymicrobial community

Origin	*Enterococcus faecalis*	*Klebsiella pneumoniae*
Patient 92	EF92-10[a]	KP92-10[b]
	EF92-11	KP92-11
Patient100	EF100-10	KP100-10
	EF100-11	KP100-11

[a]EF represents the *E. faecalis* species. EF92-10 represents the *E. faecalis* isolate collected from the 11th clinical visit of Patient 92.

[b]KP represents the *K. pneumoniae* species. KP92-10 represents the *K. pneumoniae* isolate collected from the 11th clinical visit of Patient 92.

Table S16. Two pairs of *E. faecalis* and *P. aeruginosa* strains from Patient 132 for studying bacterial interactions in polymicrobial community

Origin	*Enterococcus faecalis*	*Pseudomonas aeruginosa*
Patient 132	EF132-00[a]	PA132-00[b]
	EF132-01	PA132-01

[a]EF represents the *E. faecalis* species. EF132-00 represents the *E. faecalis* isolate collected from the first clinical visit of Patient 132.

[b]PA represents the *P. aeruginosa* species. PA132-00 represents the *P. aeruginosa* isolate collected from the first clinical visit of Patient 132.

Table S17. Statistical analysis results (p-values) for the growth *E. faecalis* and *E. coli* strains from Patients 132 and 92 in single and mixed cultures.

Patient	Species	Strain	P value	Significance level
Patient 132	*E. faecalis* (Ef)	OG1RF	0.0002	***
		132-0	<0.0001	****
		132-1	<0.0001	****
		132-2	<0.0001	****
		132-3	<0.0001	****
		132-4	<0.0001	****
		132-5	<0.0001	****
		132-6	<0.0001	****
		132-7	<0.0001	****
		132-8	<0.0001	****
		132-9	<0.0001	****
		132-10	<0.0001	****
		132-11	<0.0001	****
	E. coli (Ec)	UTI89	0.7698	ns
		132-0	0.3252	ns
		132-1	0.2381	ns
		132-2	0.9976	ns
		132-3	0.0831	ns
		132-4	0.5985	ns
		132-5	0.3110	ns
		132-6	0.2155	ns
		132-7	0.9720	ns
		132-8	0.3821	ns
		132-9	0.5719	ns
		132-10	0.1359	ns
		132-11	0.8027	ns
Patient 92	*E. faecalis* (Ef)	OG1RF	0.0002	***
		92-1	0.0004	***
		92-2	0.0003	***
		92-5	0.0007	***
		92-6	<0.0001	****
		92-7	<0.0001	****
		92-8	<0.0001	****
		92-9	<0.0001	****
		92-10	0.0002	***
		92-11	0.0005	***
	E. coli (Ec)	UTI89	0.7698	ns
		92-1	0.1857	ns
		92-2	0.0636	ns
		92-5	0.0662	ns
		92-6	0.7206	ns
		92-7	0.7578	ns
		92-8	0.1415	ns
		92-9	0.0006	***
		92-10	0.0016	**
		92-11	0.5004	ns
