# Supplementary material for: Microbial co-occurrences on catheters from long-term catheterized patients
Source: Nat Commun. 2024 Jan 2;15:61. doi: 10.1038/s41467-023-44095-0 (PMC10762172; doi:10.1038/s41467-023-44095-0)
Supplement: Supplementary file 3 — Reporting Summary [file 41467_2023_44095_MOESM3_ESM.pdf]

nature portfolio

Corresponding author(s): Scott J. Hultgren

Last updated by author(s): Nov. 20, 2023

Reporting Summary

Nature Portfolio wishes to improve the reproducibility of the work that we publish. This form provides structure for consistency and transparency in reporting. For further information on Nature Portfolio policies, see our Editorial Policies and the Editorial Policy Checklist.

Please do not complete any field with "not applicable" or n/a. Refer to the help text for what text to use if an item is not relevant to your study.
For final submission: please carefully check your responses for accuracy; you will not be able to make changes later.

Statistics

For all statistical analyses, confirm that the following items are present in the figure legend, table legend, main text, or Methods section.

n/a	Confirmed	
☐	☒	The exact sample size (*n*) for each experimental group/condition, given as a discrete number and unit of measurement
☒	☐	A statement on whether measurements were taken from distinct samples or whether the same sample was measured repeatedly
☐	☒	The statistical test(s) used AND whether they are one- or two-sided<br>*Only common tests should be described solely by name; describe more complex techniques in the Methods section.*
☒	☐	A description of all covariates tested
☒	☐	A description of any assumptions or corrections, such as tests of normality and adjustment for multiple comparisons
☐	☒	A full description of the statistical parameters including central tendency (e.g. means) or other basic estimates (e.g. regression coefficient) AND variation (e.g. standard deviation) or associated estimates of uncertainty (e.g. confidence intervals)
☐	☒	For null hypothesis testing, the test statistic (e.g. *F*, *t*, *r*) with confidence intervals, effect sizes, degrees of freedom and *P* value noted<br>*Give P values as exact values whenever suitable.*
☒	☐	For Bayesian analysis, information on the choice of priors and Markov chain Monte Carlo settings
☒	☐	For hierarchical and complex designs, identification of the appropriate level for tests and full reporting of outcomes
☒	☐	Estimates of effect sizes (e.g. Cohen's *d*, Pearson's *r*), indicating how they were calculated

Our web collection on statistics for biologists contains articles on many of the points above.

Software and code

Policy information about availability of computer code

Data collection	Catheter imagine was performed on the Odyssey Imaging System (LI-COR Biosciences) and merged in Adobe Photoshop.
Data analysis	All basic plots were generated in R v4.2.1 using Rstudio v2022.12.0+353, with the scripts to plot the data available upon request. For the co-occurrence analysis, all p-values were calculated using the cooccur package in R. For the PCoA, the dissimilarity matrix was generated using the ‘vegdist’ function from the Vegan package in R using the default settings which was passed to the built in ‘cmdscale’ function. The species scores were generated using the ‘add.spec.scores’ function from the BiodiversityR library. Comparisons of bacterial growth (measured in colony forming units, CFUs) from mono- and mixed-cultures were conducted using an unpaired t-test in GraphPad Prism 9.0 (GraphPad software).

For manuscripts utilizing custom algorithms or software that are central to the research but not yet described in published literature, software must be made available to editors and reviewers. We strongly encourage code deposition in a community repository (e.g. GitHub). See the Nature Portfolio guidelines for submitting code & software for further information.

Data

Policy information about availability of data

All manuscripts must include a data availability statement. This statement should provide the following information, where applicable:

- Accession codes, unique identifiers, or web links for publicly available datasets
- A description of any restrictions on data availability
- For clinical datasets or third party data, please ensure that the statement adheres to our policy

The data for species identification is available in the Source Data file. Sequencing files for isolates sequenced after January 14, 2020 are available at BioProject ID PRJNA956906. Despite exhaustive efforts to locate original sequencing prior to this date, the authors were unable to locate the original files. All analyses have been performed on the subset of data for which the sequencing data exists (see Supplementary Figures 8 and 9) and the conclusions, including the average species identified per patient, species identified per collection period, and relative species abundance were the same.

Research involving human participants, their data, or biological material

Policy information about studies with human participants or human data. See also policy information about sex, gender (identity/presentation), and sexual orientation and race, ethnicity and racism.

Reporting on sex and gender	Sex has been reported for de-identified patients in this study as it is an important consideration in the study and evaluation of urinary tract infections.
Reporting on race, ethnicity, or other socially relevant groupings	Race was reported in the patient demographics section.
Population characteristics	We collected catheter and urine samples approximately monthly for up to 12 collection periods from enrolled non-hospitalized, long-term catheterized patients at the Barnes-Jewish Hospital System that were over 18 years of age. The average age at the time of enrollment was 64 years and over 70% of participants were prescribed at least one course of antibiotics over the study period. The population included 25 females and 30 males.
Recruitment	Division of Urology clinic schedules were reviewed for patients meeting study eligibility criteria. Potential participants were presented with study information and informed consent at the time of their standard of care office visit by a study team member. Subjects were encouraged to review the consent form at home and contact study team for any questions. Contact was made with the patient at the time of their subsequent office visit and participation was discussed again. If willing, subjects were consented and study activities commenced.<br><br>Inclusion criteria included patients that were scheduled to undergo removal of indwelling urinary device(s) (such as catheters, both urethral and suprapubic, ureteral stents, etc.), being 18 years of age or older at the time of consent and being willing and able to provide informed consent. There were no exclusion criteria.
Ethics oversight	This study was approved by the Washington University School of Medicine (WUSM) Internal Review Board (approval #201410058) and performed in accordance with WUSM's ethical standards and the 1964 Helsinki declaration and its later amendments.

Note that full information on the approval of the study protocol must also be provided in the manuscript.

Field-specific reporting

Please select the one below that is the best fit for your research. If you are not sure, read the appropriate sections before making your selection.

☒ Life sciences ☐ Behavioural & social sciences ☐ Ecological, evolutionary & environmental sciences

For a reference copy of the document with all sections, see nature.com/documents/nr-reporting-summary-flat.pdf

Life sciences study design

All studies must disclose on these points even when the disclosure is negative.

Sample size	55 long-term catheterized patients were included in this study totaling 366 catheter/urine samples which far exceeds patient enrollment in comparable studies. Sample sizes were chosen to maximize the number of participants and collection periods over the enrollment period.
Data exclusions	Data were not excluded from this study.
Replication	Were applicable, experiments were performed in at least biological triplicate and the standard deviations were reported. All attempts at replication were successful.

Randomization	Randomization is not applicable to this study. The study was observational, there were no control or treatment groups. The co-occurrence analysis was based on a probabilistic model that did not require randomization.
Blinding	Blinding is not applicable to this study as the study was observational, there were no control or treatment groups.

Reporting for specific materials, systems and methods

We require information from authors about some types of materials, experimental systems and methods used in many studies. Here, indicate whether each material, system or method listed is relevant to your study. If you are not sure if a list item applies to your research, read the appropriate section before selecting a response.

Materials & experimental systems

n/a	Involved in the study	
☐	☒	Antibodies
☒	☐	Eukaryotic cell lines
☒	☐	Palaeontology and archaeology
☒	☐	Animals and other organisms
☒	☐	Clinical data
☒	☐	Dual use research of concern
☒	☐	Plants

Methods

n/a	Involved in the study	
☒	☐	ChIP-seq
☒	☐	Flow cytometry
☒	☐	MRI-based neuroimaging

Antibodies

Antibodies used	Enterococcus polyclonal antibody (PA1-73120, Invitrogen) , E. coli serotype O/K Polyclonoal Antibody (PA1-73032, Invitrogen), IRDye 680LT Donkey anti-Rabbit (LI-COR, 926-68023) , IRDye 800CW Donkey-anti Goat (LI-COR, 926-32214)
Validation	All primary and secondary antibodies used are commercially available. Validation statements for use of primary antibodies in immunofluorescence is available on the company website and have been detailed in the Methods: Enterococcus polyclonal antibody (PA1-73120, Invitrogen, validation statement for immunofluorescence application available at: https://www.thermofisher.com/antibody/product/Enterococcus-Antibody-Polyclonal/PA1-73120) and E. coli serotype O/K Polyclonoal Antibody (PA1-73032, Invitrogen, validation statement for immunofluorescence application available at https://www.thermofisher.com/antibody/product/E-coli-serotype-O-K-Antibody-Polyclonal/PA1-73032).

This checklist template is licensed under a Creative Commons Attribution 4.0 International License, which permits use, sharing, adaptation, distribution and reproduction in any medium or format, as long as you give appropriate credit to the original author(s) and the source, provide a link to the Creative Commons license, and indicate if changes were made. The images or other third party material in this article are included in the article's Creative Commons license, unless indicated otherwise in a credit line to the material. If material is not included in the article's Creative Commons license and your intended use is not permitted by statutory regulation or exceeds the permitted use, you will need to obtain permission directly from the copyright holder. To view a copy of this license, visit http://creativecommons.org/licenses/by/4.0/
